# An Investigation into the Evolutionary Characteristics and Expression Patterns of the Basic Leucine Zipper Gene Family in the Endangered Species *Phoebe bournei* Under Abiotic Stress Through Bioinformatics

**DOI:** 10.3390/plants14152292

**Published:** 2025-07-25

**Authors:** Yizhuo Feng, Almas Bakari, Hengfeng Guan, Jingyan Wang, Linping Zhang, Menglan Xu, Michael Nyoni, Shijiang Cao, Zhenzhen Zhang

**Affiliations:** 1 College of Forestry, Fujian Agriculture and Forestry University, Fuzhou 350002, China; 15532106788@163.com (Y.F.); almasbakari154@gmail.com (A.B.); g1290570831@163.com (H.G.); 2 Fujian Provincial Key Laboratory of Haixia Applied Plant Systems Biology, Synthetic Biology Center, Haixia Institute of Science and Technology, Fujian Agriculture and Forestry University, Fuzhou 350002, China; jingyanwang0313@163.com (J.W.); 18058302106@163.com (L.Z.); 15673123482@163.com (M.X.); 3 College of Life Sciences, Fujian Agriculture and Forestry University, Fuzhou 350002, China; 4 Department of Agrifood, Environmental and Animal Sciences, Università degli Studi di Udine, Via Palladio, 8, 33100 Udine, UD, Italy; michaelnyoni.mn@gmail.com

**Keywords:** abiotic stresses, expression analysis, *Phoebe bournei*, *bZIP* gene family

## Abstract

The *bZIP* gene family play a crucial role in plant growth, development, and stress responses, functioning as transcription factors. While this gene family has been studied in several plant species, its roles in the endangered woody plant *Phoebe bournei* remain largely unclear. This study comprehensively analyzed the *PbbZIP* gene family in *P. bournei*, identifying 71 *PbbZIP* genes distributed across all 12 chromosomes. The amino acid count in these genes ranged from 74 to 839, with molecular weights varying from 8813.28 Da to 88,864.94 Da. Phylogenetic analysis categorized the *PbbZIP* genes into 12 subfamilies (A-K, S). Interspecific collinearity analysis revealed homologous *PbbZIP* genes between *P. bournei* and *Arabidopsis thaliana*. A promoter cis-acting element analysis indicated that *PbbZIP* genes contain various elements responsive to plant hormones, stress signals, and light. Additionally, expression analysis of public RNA-seq data showed that *PbbZIP* genes are distributed across multiple tissues, exhibiting distinct expression patterns specific to root bark, root xylem, stem bark, stem xylem, and leaves. We also performed qRT-PCR analysis on five representative *PbbZIP* genes (*PbbZIP14*, *PbbZIP26*, *PbbZIP32*, *PbbZIP67*, and *PbbZIP69*). The results demonstrated significant differences in the expression of *PbbZIP* genes under various abiotic stress conditions, including salt stress, heat, and drought. Notably, *PbbZIP67* and *PbbZIP69* exhibited robust responses under salt or heat stress conditions. This study confirmed the roles of the *PbbZIP* gene family in responding to various abiotic stresses, thereby providing insights into its functions in plant growth, development, and stress adaptation. The findings lay a foundation for future research on breeding and enhancing stress resistance in *P. bournei*.

## 1. Introduction

Adverse environmental factors, such as drought, salinity, and extreme temperatures, significantly impact plant growth and yield [1]. To cope with these stresses, plants have evolved complex defense mechanisms, including signaling pathways mediated by receptor-like kinases (RLKs), phosphatases, protein kinases, and transcription factors [2]. RLKs play a crucial role in initiating downstream signaling cascades. They regulate gene expression and metabolic processes, enabling plants to adapt to and survive under adverse conditions [3]. In the signaling pathways, protein phosphorylation acts as a molecular switch, modulating the activity of various proteins associated with stress responses [4]. In addition, transcription factors enhance plant tolerance to harsh environments by coordinating the expression of stress-responsive genes [5,6].

The basic leucine zipper (*bZIP*) transcription factor family is widely found in eukaryotes. bZIP proteins consist of two domains: a basic region (N-x7-R/K-x9) for sequence-specific DNA binding and a leucine zipper domain for protein dimerization [7]. This unique structure allows *bZIP* transcription factors to form homodimers or heterodimers with other transcription factors. They can then recognize and bind to specific cis-acting elements in the promoters of target genes, such as the ACGT core sequence (A-box, C-box, and G-box), and regulate gene expression accordingly [8,9]. Researchers have successfully identified members of the *bZIP* transcription factor family in various plant species, including *Arabidopsis thaliana* (L.) [9], *Oryza sativa* L. [7], *Zea mays* L. [10], *Juglans regia* L. [11], *Populus* L. [12], *Ziziphus jujuba* Mill. [13], and *Carthamus tinctorius* L. [14]. As indicated by numerous studies, putative *bZIP* genes are categorized into several groups based on the sequence similarity of their basic regions and conserved motifs. While the *AtbZIP* gene family in *A. thaliana* is classified into 13 subfamilies, the bZIP proteins in rice are divided into 11 groups based on DNA-binding specificity and amino acid sequence prediction. Despite the difference in the number of groups within the *bZIP* gene family between rice (11 groups) and *A. thaliana* (13 groups), the membership of these groups shows similarity. *bZIP* transcription factors are crucial regulatory elements in plants’ responses to abiotic stresses such as salt, drought, and temperature [15,16,17,18]. For example, studies on *O. sativa* and *A. thaliana* have demonstrated that specific *bZIP* genes are up-regulated in response to salt stress, thereby enhancing stress tolerance through ion homeostasis and antioxidant mechanisms [19,20]. In *Arabidopsis*, *bZIP37* (ABF3) directly induces the expression of late embryo abundant (*LEA*) genes by participating in stomatal closure and reprogramming metabolic processes. It also accumulates intracellular protective osmoregulatory substances, thereby protecting cellular water supply, reducing water evaporation, and adapting to drought-stressed environments [21].

Additionally, the subcellular localization of *bZIPs* is also regulated by abiotic stress. For example, it has been shown that bZIP52 and bZIP18 play a significant repressive role in the nucleus, and their mutations result in nearly twice as many down-regulated differentially expressed genes as up-regulated differentially expressed genes [22]. Arabidopsis bZIP52 and bZIP18 proteins are dephosphorylated, rendering them incapable of interacting with 14-3-3 proteins and causing their dissociation and subsequent translocation to the nucleus. This enables them to enter the nucleus and co-regulate the expression of many genes, thereby adapting to changes in the heat stress environment [22]. Additionally, bZIP transcription factors from *A. thaliana*, including bZIP16, bZIP68, and GBF1, are reported to regulate the formation of photosynthetically active chloroplasts in response to light. These proteins modulate gene expression that is critical for plant development across various environmental conditions. Notably, their function is influenced by the redox state of a conserved cysteine residue, acting as a molecular switch for regulating light-responsive genes [23].

*Phoebe bournei* (Hemsl.), a precious evergreen tree in the Lauraceae family, is distributed across southern Chinese provinces, including Fujian, Jiangxi, and Guangdong [24]. Its high-quality timber is used in furniture, construction, and traditional Chinese medicine [25,26]. However, it is now endangered due to over-exploitation and habitat loss, prompting significant conservation and afforestation efforts [27,28]. Like many plants, it faces various abiotic stresses, including drought, extreme temperatures, and salinity [29,30,31,32,33]. For example, salt stress can significantly reduce plant growth, productivity, and survival rates through the effects of ion toxicity, osmotic stress, and oxidative damage. To adapt to these conditions, plants must activate complex internal regulatory networks, including transcriptional-level changes [34,35,36].

Given the key role of *bZIP* genes in plant abiotic stress tolerance, studying the *P. bournei bZIP* gene family is highly significant. It can clarify the molecular mechanisms of stress resistance, offering theoretical support for conservation and breeding. Identifying and analyzing this gene family can also reveal its functions in growth, development, and stress responses, providing valuable genetic resources to enhance *P. bournei*’s stress tolerance.

This study aims to identify *P. bournei bZIP* transcription factors and analyze their gene structure, chromosomal location, duplication events, expression patterns, and response to abiotic stresses (salt, high temperature, and osmotic stress). It will also compare them with *Arabidopsis bZIP* genes to explore their evolutionary and functional relationships across species. The findings will provide a theoretical basis for enhancing *P. bournei*’s tolerance to abiotic stress and offer valuable resources for its breeding and conservation strategies.

## 2. Materials and Methods

### 2.1. Plant Material, Data Sources, and Conditions for Growth

The genome sequence data and annotation information of *P. bournei* were downloaded from the sequence archive of the China National Gene Bank Database (Accession Number: CNA0029376 (https://db.cngb.org/search/assembly/CNA0029376/ (accessed on 4 May 2024))). The *A. thaliana* genome sequence file was obtained from EnsemblPlants (https://plants.ensembl.org/Arabidopsis_thaliana/Info/Index/ (accessed on 4 May 2024)). We conducted the experiments at the Fujian Academy of Forestry in China, where the seedlings were grown outdoors for ten months in red soil with a pH of 5 and a soil organic matter content of 2.57% to 6.07%. The growing area had an average annual temperature of 16–20 °C, with an average yearly precipitation of 900–2100 mm and a relative humidity of about 77%.

### 2.2. The PbbZIP Gene Identification and Physicochemical Characteristics

To identify the *bZIP* gene family members in *P. bournei*, we first obtained the protein sequences of *A. thaliana bZIP* genes from the TAIR database (https://www.arabidopsis.org/ (accessed on 6 May 2024)). Subsequently, using the TBtools-II v 2.10 software, we accurately extracted CDS sequences from the genome annotation information of *P. bournei* and simplified them into protein files. Then, we compared the protein sequences of the *bZIP* gene family from *A. thaliana* with the corresponding sequences of *P. bournei* and conducted a BLASTP search on NCBI to consult the annotation information of *P. bournei*. We then preliminarily screened out the bZIP family protein sequences of *P. bournei*. To further verify and identify *bZIP* gene family members, we downloaded the HMM of the bZIP conserved domain (PF00170, PF03131, PF07716) from the Pfam database and used HMMER-3.2.1 (https://www.ncbi.nlm.nih.gov/Structure/bwrpsb/bwrpsb.cgi (accessed on 8 May 2024)) for the search. The expected value was set to less than 10^−5^, and all other parameters were kept as their default values. By comparing the protein sequences obtained via BLASTp and HMMER, we selected the consistent sequences for subsequent analysis [37,38]. The physical and chemical properties of the identified *bZIP* gene family were calculated using the ExPASy website (https://www.expasy.org/ (accessed on 10 May 2024)), with indicators such as isoelectric point, number of amino acid residues, and molecular weight. Subcellular localization prediction analysis was also performed using the WOLF PSORT website (https://wolfpsort.hgc.jp/ (accessed on 10 May 2024)) [39].

### 2.3. PbbZIP Gene Family Chromosomal Distribution

Chromosomal distribution and gene density information for *bZIP* family members were extracted from the *P. bournei* GFF file using TBtools-IIv2.10, and a chromosomal distribution map of *bZIP* genes was constructed.

### 2.4. Construction and Analysis of Evolutionary Tree and Collinearity Analysis

To gain an in-depth understanding of the differences and evolutionary relationships among *bZIP* sequences across various species, this study utilized MEGA7.0 software for a comparative analysis of *bZIP* sequences from *P. bournei* and *A. thaliana*. During the process, default parameters were applied, with the bootstrap value set to 1000 replicates. The neighbor-joining method and p-distance model were selected for constructing the phylogenetic tree, ensuring the reliability of the findings. Subsequently, we made changes to the phylogenetic tree and assigned nodes using the Evolview website (https://evolgenius.info/evolview-v2/ (accessed on 28 August 2024)) for a more intuitive and clear presentation of the research outcomes. The TBtools program was used to determine their relationship. The plant genome database provided the whole-genome sequences and gene annotation files of two species, which were used to identify the standard features of the homologous *bZIP* gene family in P. borynei and *A. thaliana*. The Commonality Analysis Atlas was created using TBtools-v2.10 software. To examine selection pressure, we used the Ka/Ks_Calculator tool within TBtools to calculate the nonsynonymous substitution rate (Ka), the synonymous substitution rate (Ks), and their ratio (Ka/Ks). The calculation utilized the YN model implemented in the Ka/Ks_Calculator, with a threshold of Ka/Ks = 1 set to indicate positive selection [40]. TBtools was used for grepping the chromosomal location information of the *PbbZIP* genes from the genome (FASTA) file and the annotation (GFF) file of *P. bournei*. Gene duplication and syntenic relationships of *PbbZIP* were determined using MCScanX (https://smart.embl.de/ (accessed on 12 July 2025)) with default parameters and plotted using TBtools-v2.10.

### 2.5. Motif Analysis and Gene Structure of the PbbZIPs

We found the conserved motifs in PbbZIP proteins using the online tool MEME Suite 5.4.1 (http://meme-suite.org/ (accessed on 28 August 2024)). The maximum number of motifs was set to 25, and the motif sites corresponded to a ZOOPS (Zero or One Occurrence Per Sequence) model [41]. GFF (Generic Feature Format) annotation files from the *P. bournei* genome were used to determine the intron–exon location of the *PbbZIP* genes. TBtools were used to visualize the gene structure and motifs [42].

### 2.6. Examination of Cis-Elements in the Promoters of PbbZIP Genes

In *P. bournei*, TBtools was used to extract the promoter sequences of *bZIP* genes, where the sequence 2000 bp upstream of the transcription start site was designated as the target for analysis. These promoter sequences were then submitted to the PlantCARE website (http://bioinformatics.psb.ugent.be/webtools/plantcare/html, accessed on 28 August 2024) for analysis. The output data underwent filtering and processing, during which the exact start and end positions of the promoters were determined. Finally, TBtools was employed once more to visualize the analysis results.

### 2.7. Transcriptome Data Acquisition and Abiotic Stress Treatment of Different Plant Tissues

In this study, we searched the *P. bournei* gene PRJNA628065 in the European Bioinformatics Institute (EBI) database (https://www.ebi.ac.uk (accessed on 28 August 2024) and successfully retrieved transcriptomic expression data for five different tissues of *P. bournei*. The TBtools were used to evaluate gene expression data and create a gene expression heat map using annual seedlings purchased from the Fujian Academy of Forestry. The seedlings were cultivated in an artificial climate box with various treatments. *P. bournei* seedlings with comparable growth potential were selected for treatment. These *P. bournei* seedlings were then subjected to specific treatment regimens. Among them, three *P. bournei* seedlings were designated as the control group, while the remaining materials were divided into two groups: a control group and a stress treatment group. After the treatments, leaf samples were collected and stored in liquid nitrogen at −80 °C for RNA extraction. The seedlings in the treatment group were soaked in a nutrient solution containing 10% PEG to simulate drought. The control group seedlings were soaked in distilled water. A 10% NaCl nutrient solution was applied to the group receiving the salt treatment. For temperature treatment, the treatment group was incubated at 40 °C, while the control group remained at room temperature. All samples were incubated in an artificial climate incubator set at a temperature of 25 °C and a relative humidity of 75%. The control group was sampled at 0 h, and the treatment groups were sampled at 6 h, 12 h, and 24 h.

### 2.8. Abiotic Stress Experiment and qRT-PCR Analysis

RNA was extracted from the collected plant samples, and the levels of target gene expression were monitored using a quantitative reverse transcriptase PCR (RT-qPCR) assay. A heat map labeled with correlation clusters was created using the Spearman correlation algorithm to represent the relationships between patterns of gene expression [43]. Total RNA was extracted from both the stress-treated samples and the control samples using an RNA extraction kit (Omega Bio-TEK, Shanghai, China). According to the manufacturer’s instructions, cDNA was synthesized using EasyScript^®^ One-Step gDNA Removal and cDNA Synthesis SuperMix (Transgen, Beijing, China). TransStart^®^ top green qPCR SuperMix (Transgen, Beijing, China) was used for RT-qPCR. The internal reference gene used was PbEF1α (GenBank No. KX682032), and Table 1 lists the primers used. The comparative delta–delta Ct approach was utilized to determine the relative transcript levels. Subsequently, a one-way ANOVA, accompanied by Duncan’s multiple range tests at a 95% confidence interval, was performed using the GraphPad Prism 10.12 software. (https://www.graphpad.com/ (accessed on 28 August 2024)) [44,45]. Three biological replicates and three technical replicates are used for each quantitative PCR [46].

## 3. Results

### 3.1. Identification and Physicochemical Characteristics of the PbbZIP Gene

According to the results, 71 *PbbZIP* genes were identified. *PbbZIP1* was renamed to *PbbZIP71* due to its gene distribution on chromosome 12 of *P. bournei*. Among these, the physical and chemical properties are integrated, as shown in Table 2. The number of amino acids ranged from 74 residues in PbbZIP15 to 839 residues in PbbZIP71, and the relative molecular weight varied from 8813.28 Da (PbbZIP15) to 88,864.94 Da (PbbZIP71). The theoretical isoelectric point (PI) was between 4.78 (PbbZIP26) and 11.45 (PbbZIP48). Based on the principle that amphiphilic proteins have a hydrophilicity index between −0.5 and +0.5 (negative values of the Grand Average of Hydropathy, GRAVY, indicate hydrophilicity, and positive values indicate hydrophobicity), it was found that the entire *PbbZIP* gene family showed negative GRAVY values, suggesting that most of them were amphiphilic proteins (Table 2). In addition, the predicted subcellular localizations of all the PbbZIPs were expected to be in the nucleus.

### 3.2. Chromosomal Localization of bZIP Genes in P. bournei

**Figure 1:**
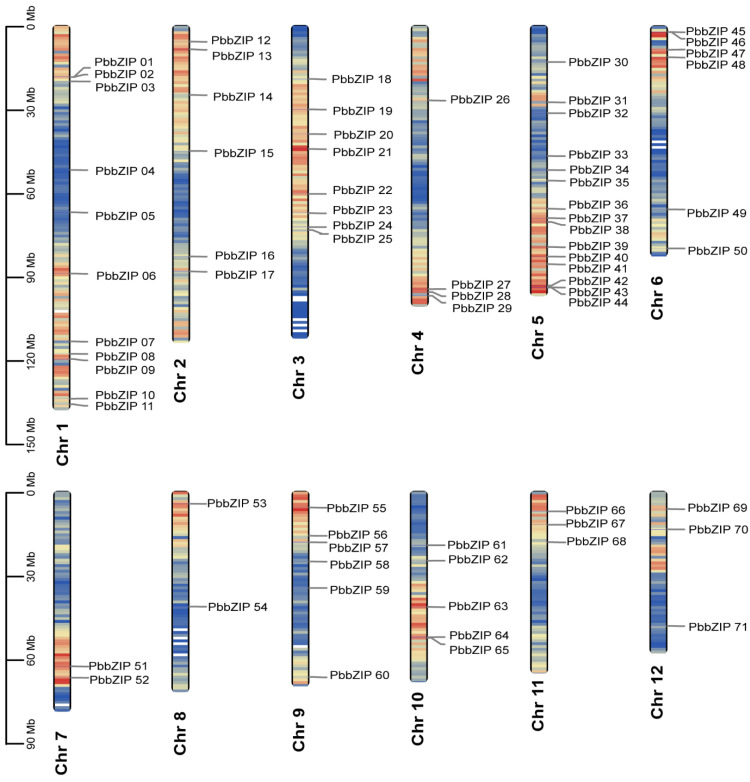
Chromosomal location of the identified *PbbZIP* genes in *P. bournei*. The chromosomal location of the 71 mapped *PbbZIP* genes is depicted from top to bottom. The scale bar is in megabases (Mb). Chromosome numbers are counted from the left side of the corresponding chromosomes. The colors red and blue indicate gene distribution within a chromosome, with red signifying a high distribution.

The chromosomal locations of the 71 *PbbZIP* genes identified in *P. bournei* are depicted in Figure 1. All observed genes were mapped to all 12 chromosomes, but were not evenly distributed. Each chromosome contained two to fifteen *PbbZIP* genes. For instance, chromosomes 7 and 8 had the fewest number of genes, with only two genes each: *PbbZIP51* and *PbbZIP52* on chromosome 7, and *PbbZIP53* to *PbbZIP54* on chromosome 8. In contrast, chromosome 5 had the most significant number of genes, ranging from *PbbZIP30* to *PbbZIP44*. Additionally, chromosomes 1, 2, 3, 6, and 9 had more than five *PbbZIP* genes, respectively, with from *PbbZIP1* to *PbbZIP11* on chromosome 1, from *PbbZIP12* to *PbbZIP17* on chromosome 2, from *PbbZIP18* to *PbbZIP25* on chromosome 3, from *PbbZIP45* to *PbbZIP50* on chromosome 6, and from *PbbZIP55* to *PbbZIP60* on chromosome 9. Furthermore, *PbbZIP26* to *PbbZIP29* were located on chromosome 4. *PbbZIP61* to *PbbZIP65* were situated on chromosome 10, and *PbbZIP66* to *PbbZIP68* were positioned on chromosome 11, while *PbbZIP69* to *PbbZIP71* were positioned on chromosome 12. Such a distribution may be conducive to the functional diversification and synergistic interactions among the members of the gene family.

### 3.3. Multiple Sequence Alignment Analysis of the PbbZIP Gene Family

**Figure 2:**
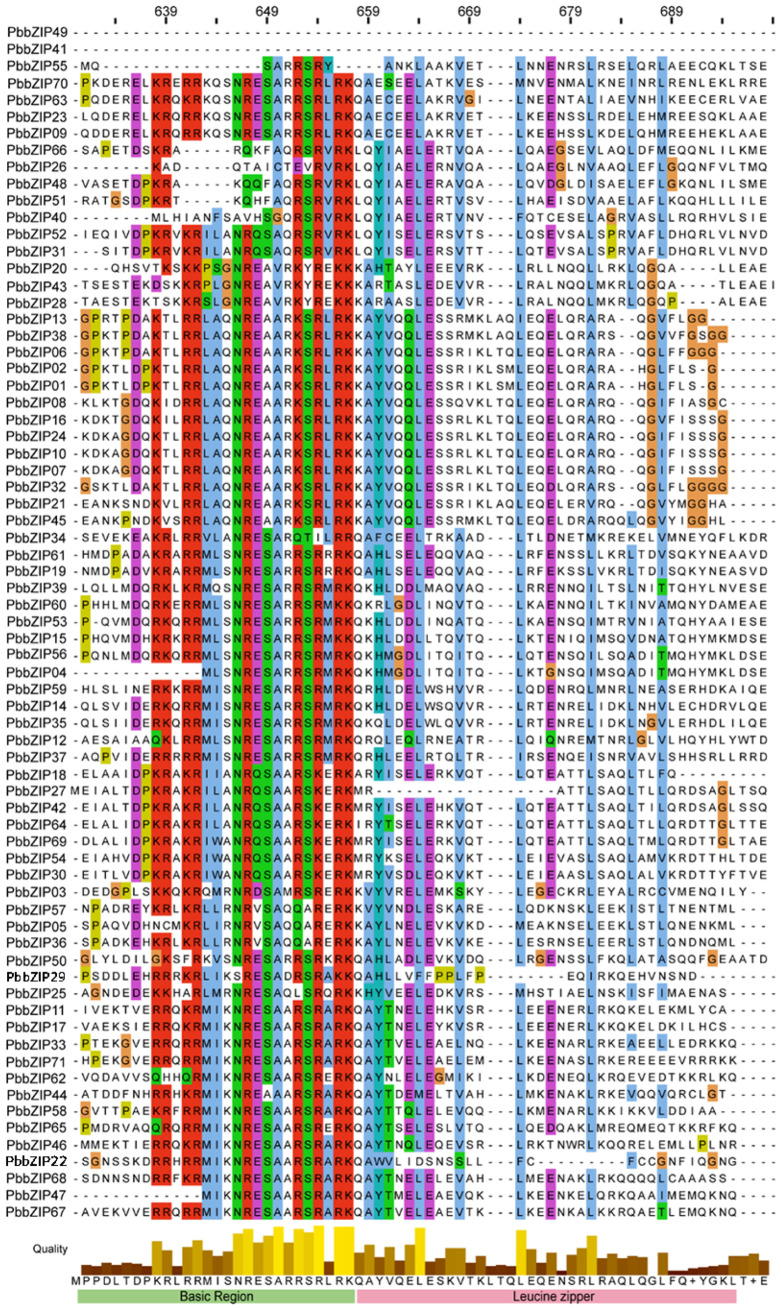
The multiple sequence alignment of the DNA binding domain in the PbbZIP protein sequence using Jalview-2.11.3.0 software.

To further clarify the evolutionary relationships of *bZIP*s, we also conducted a multiple sequence alignment analysis. As demonstrated by our Pfam and SMART analyses, the 71 *P. bournei* proteins all possessed the bZIP domain, which was in line with our initial hypothesis. Consequently, after translating them into the amino acid sequences of each member from the bZIP-conserved domain [47,48], we carried out multiple sequence alignments. The results are displayed in Figure 2. The bZIP domain comprises a fundamental DNA-binding region and an adjacent leucine zipper structure. The NRxSA[R/K]RSRxRK motif is consistently present within the basic area. Intriguingly, a comparable conserved motif, “RHx[R/H]SxS”, has been recently reported in *Arabidopsis* and demonstrated to be a conserved phosphorylation motif [49]. Meanwhile, the leucine zipper domain consists of a heptapeptide repeat of leucine (L) or a related hydrophobic amino acid. These domains were also found in *P. bournei* bZIP proteins. 

### 3.4. Phylogenetic and Co-Linearity Analysis of the PbbZIP Genes

**Figure 3:**
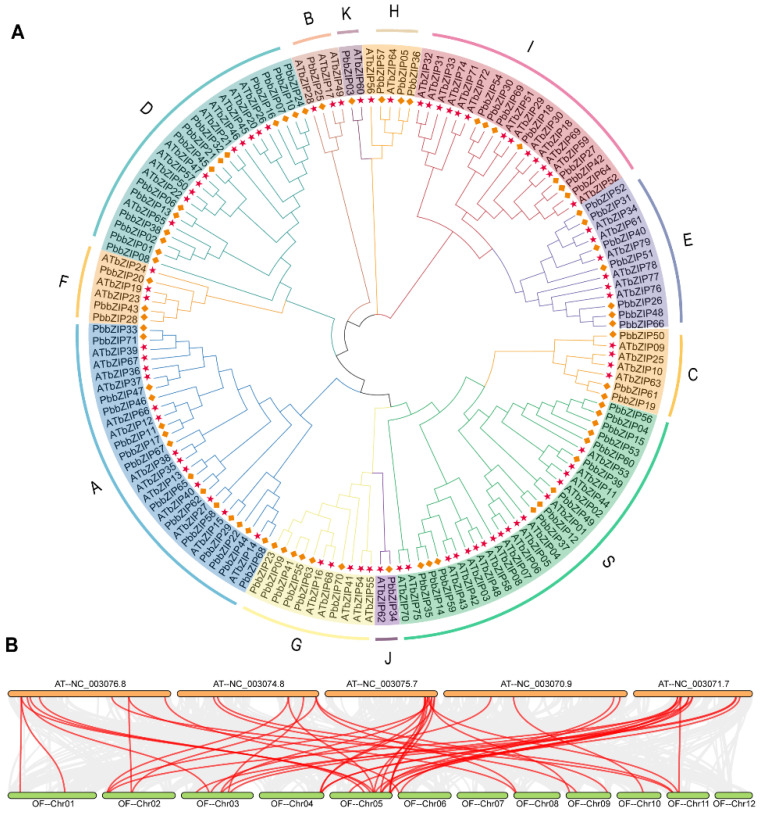
(**A**) Phylogenetic analysis of complete bZIP protein sequences from *P. bournei* (Pb) and *A. thaliana* (At). The number on the branch signifies the reliability of the node based on 1000 iterations of bootstrap analysis. Asterisks (*) indicate strong statistical support (bootstrap value > 90) for specific nodes. Branches of different colors represent different subfamilies. Key divergence points are marked with diamonds (♦), illustrating significant evolutionary and speciation events within the *PbbZIP* gene family in response to stress conditions. (**B**) Homology analysis between *the P. bournei genome* and the *A. thaliana* genome. The gray lines represent the aligned blocks between paired genomes, and the red lines denote collinear *PbbZIP* gene pairs.

By performing multiple sequence alignments of the 78 bZIP proteins from *A. thaliana* and the 71 bZIP proteins from *P. bournei*, a maximum likelihood phylogenetic tree was constructed using MEGA7.0 to depict the affinity and evolutionary relationship between species and genes. Based on the *bZIP* gene family classification system of *A. thaliana*, the *P. bournei bZIP* gene family was divided into 12 major subgroups, namely from Group A to group K, and Group S (Figure 3A) [9]. Among them, Group A was a fairly large family, forming an independent branch and consisting of 14 group members, including *PbbZIP33*, *PbbZIP71*, *PbbZIP4*, *PbbZIP46*, *PbbZIP11*, *PbbZIP17*, *PbbZIP67*, *PbbZIP65*, *PbbZIP62*, *PbbZIP58*, *PbbZIP29*, *PbbZIP22*, *PbbZIP44*, *and PbbZIP68*. In addition, Group D (*PbbZIP38*, *PbbZIP01*, *PbbZIP02*, *PbbZIP08*, *PbbZIP13*, *PbbZIP06, PbbZIP24*, *PbbZIP45*, *PbbZIP21*, *PbbZIP32*, *PbbZIP10*, *PbbZIP07*, and *PbbZIP16*) was also a relatively large group, and its members were in the same branch as Group F (*PbbZIP20*, *PbbZIP43*, and *PbbZIP28*), indicating they shared similarities. There was only one gene in Group B (*PbbZIP25*), which was positioned on a single branch by itself. Groups K (*PbbZIP03*) and H (*PbbZIP36*, *PbbZIP05,* and *PbbZIP57*) were small assemblages and possessed relatively high sequence identity. Groups I and E were large groups and were genetically conserved, including Group I with seven members (*PbbZIP64*, *PbbZIP42*, *PbbZIP27*, *PbbZIP18*, *PbbZIP69*, *PbbZIP30*, and *PbbZIP54*) and Group E with seven members (*PbbZIP66*, *PbbZIP48*, *PbbZIP26*, *PbbZIP51*, *PbbZIP40*, *PbbZIP31*, and *PbbZIP52*). Groups C, S, J, and G were situated within the same large branch, implying that they shared certain homologies. They were Group C (3 members) *PbbZIP19*, *PbbZIP61*, and *PbbZIP50*, S (12 members) *PbbZIP35*, *PbbZIP14*, *PbbZIP59*, *PbbZIP37*, *PbbZIP12*, *PbbZIP49*, *PbbZIP39*, *PbbZIP60*, *PbbZIP53*, *PbbZIP15*, *PbbZIP04*, and *PbbZIP56*, G (6 members) *PbbZIP23*, *PbbZIP09*, *PbbZIP41*, *PbbZIP55*, *PbbZIP63*, and *PbbZIP70*, and J with a single member *PbbZIP34*. To gain deeper insights our research revealed that, excluding chromosome 12, 11 of *P. bournei*’s 12 chromosomes exhibited collinearity with *A. thaliana* genes within the chromosomal position range of 003076.8 to 003071.7. Moreover, synteny analysis revealed 45 collinear gene pairs between the *P. bournei PbbZIPs* and the *A. thaliana AtbZIPs* (Figure 3B); notably, both members of each pair belong to the identical subfamily (Table 3). It suggests that the bZIP proteins of *A. thaliana* and *P. bournei* may share a common ancestor in their evolutionary history or follow similar evolutionary patterns in maintaining gene function and genomic structure.

**Figure 4:**
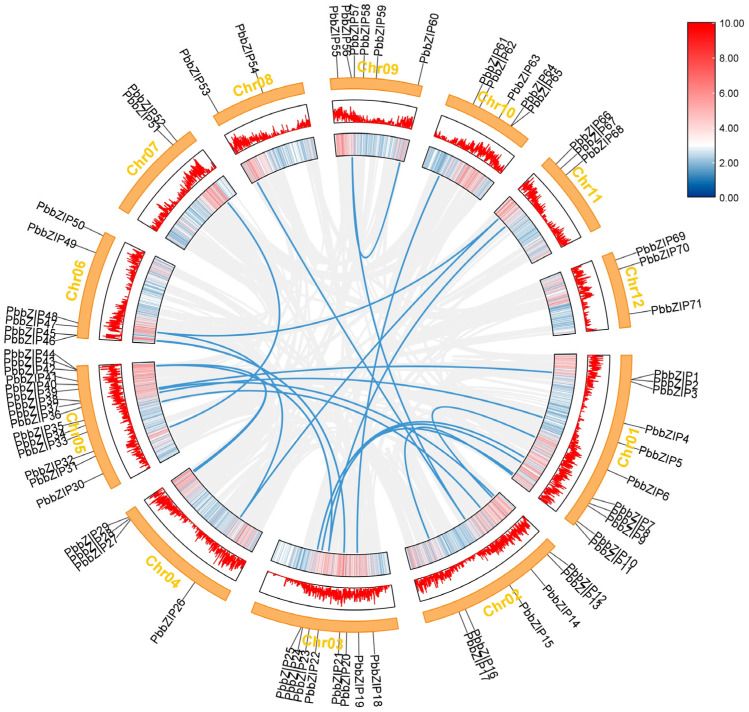
The genome map of *Phoebe bournei* is displayed as a circle. The outer segments of the circle correspond to the 12 assembled chromosomes, which are labeled sequentially from chromosome 1 (Chr01) to chromosome 12 (Chr12). Looking inward from the outermost edge of each chromosome segment, the first circle presents nucleotide positions measured in megabases (Mb), which scale the genetic map. Immediately adjacent to this is a visual display of gene density, where the peaked portions imply regions of denser genes. The gray lines in the innermost circle represent all replicated gene pairs in the *Phoebe bournei* genome, while the blue lines indicate the co-located gene pairs of *PbbZIP*.

In addition, to gain deeper insights into the evolutionary history of the *PbbZIP* gene family an intraspecific covariance analysis was performed (Figure 4). The results of this analysis revealed one tandem duplication event (involving *PbbZIP56* and *PbbZIP60*) and twenty-one segmental duplication events (including gene pairs such as *PbbZIP1* and *PbbZIP38*, *PbbZIP5* and *PbbZIP36*, *PbbZIP6* and *PbbZIP13*, *PbbZIP24* with *PbbZIP7* and *PbbZIP10*, *PbbZIP9* and *PbbZIP23*, *PbbZIP11* and *PbbZIP17*, *PbbZIP12* and *PbbZIP37*, *PbbZIP14* and *PbbZIP35*, *PbbZIP15* with *PbbZIP53* and *PbbZIP56*, *PbbZIP19* and *PbbZIP61*, *PbbZIP21* and *PbbZIP45*, *PbbZIP22* with *PbbZIP44* and *PbbZIP68*, *PbbZIP26* with *PbbZIP48* and *PbbZIP66*, *PbbZIP28* and *PbbZIP43*, *PbbZIP29* and *PbbZIP44*, *PbbZIP31* and *PbbZIP52*, *PbbZIP48* and *PbbZIP66*, and *PbbZIP56* and *PbbZIP60*). The *PbbZIP* genes in Figure 4 that were not generated by duplication events may have originated from diverse mechanisms such as gene loss, independent origination, and transposition events. Our analysis suggests that some *PbbZIP* genes may have lost their homologous genes during evolution, thereby developing unique functions. Others may have emerged independently without clear duplication events. Additionally, some *PbbZIP* genes may have migrated within the genome through transposition mechanisms, leading to the formation of new genomic locations. These findings suggest that the *PbbZIP* transcription factor family is primarily composed of genes generated by large-scale segmental duplication events, with some genes arising from small-scale tandem duplication events. Furthermore, the distribution of these duplicated genes indicates potential functional or evolutionary connections between *PbbZIP* genes located on different chromosomes.

### 3.5. Protein Motif and Exon–Intron Structure Analysis of the PbbZIP Genes

**Figure 5:**
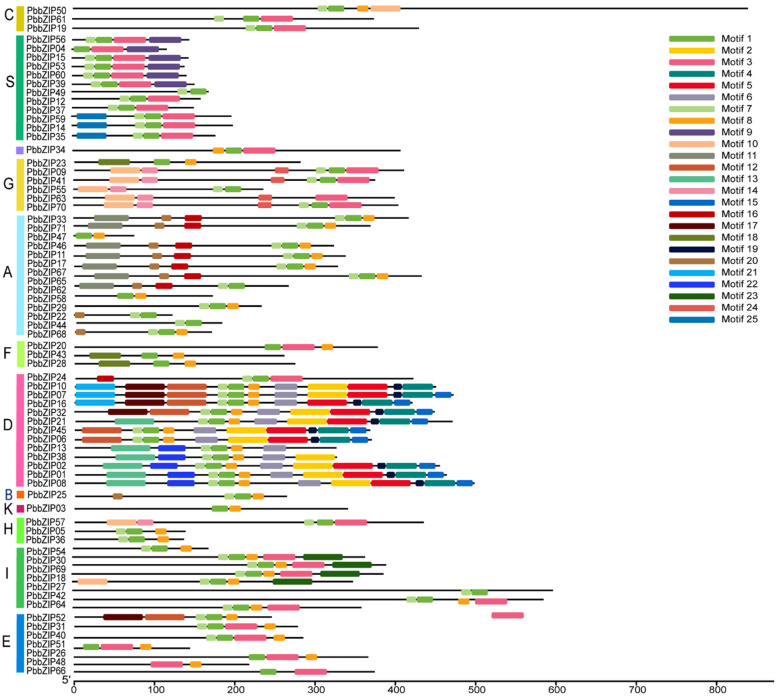
Distribution of conserved motifs of the PbbZIP protein.

To track the evolutionary footprints and disclose the features of gene structures, as well as to provide references for revealing the potential functional characteristics and action mechanisms of proteins, we conducted analyses on protein domains and intron–exon structures. Notably, members of the same subfamily showed consistent motif composition and sequential arrangement (Figure 5). Among the 71 identified members, it was demonstrated that motifs 1 and 7 tended to emerge concurrently and were present in the majority of genes (Figure 5). Group D encompassed the most incredible diversity of domains, nearly covering all the domain types. Moreover, a significant portion of the genes within this group possessed motifs such as 1, 2, 4, 5, 6, 7, 8, 11, 15, and 19 (Figure 5). The genomes of members in Group S were generally relatively small, but were rich in multiple conserved motifs, such as motif 1, motif 7, and motif 14 (Figure 5).

**Figure 6:**
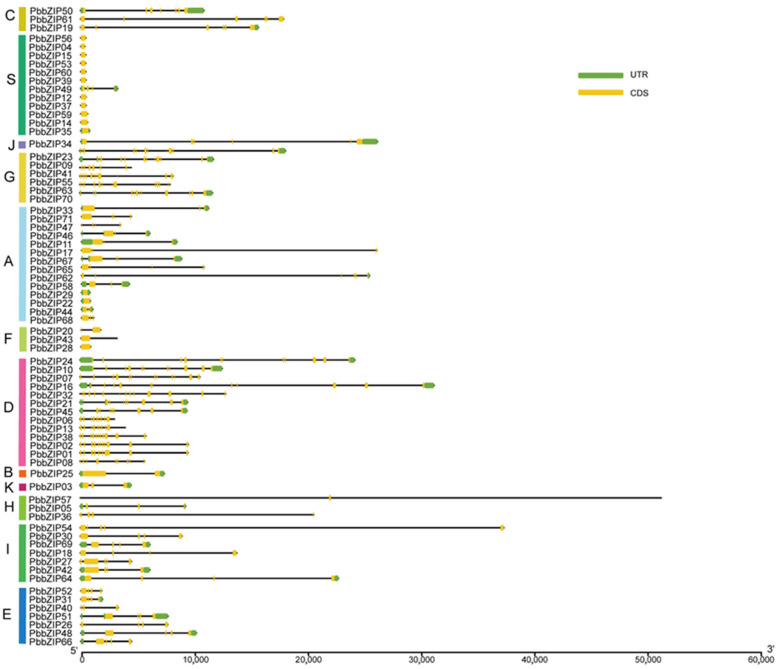
Exon–intron structure of the *PbbZIP* genes. Green boxes indicate exons (CDS), black lines indicate introns, and yellow boxes indicate 5′ and 3′ untranslated regions.

In plants, introns are known to play a crucial role in regulating gene expression [44,50]. Consequently, it is significant to elucidate gene function through the analysis of the intron–exon structure. The gene architecture of *PbbZIP* family members (Figure 6) revealed that the number of exons varied from 1 to 14, and the number of introns ranged from 3 to 13. Some genes are larger because of the presence of large introns, such as *PbbZIP54* and *PbbZIP57*. *PbbZIP17* and *PbbZIP54* lack untranslated regions (UTRs), which may be attributed to the limitations of genome annotation, whereas all other *PbbZIP* genes have 5′ UTRs. The UTR of a gene sequence is crucial for mRNA stability. Significant differences were observed in the position and number of exons, as well as the length of introns, among different family members.

### 3.6. Cis-Acting Elements Analysis of PbbZIP Genes

To elucidate the biological functions and regulatory mechanisms of *PbbZIP* genes in *P. bournei*, we conducted an in-depth analysis of the promoter regions of these genes, with a focus on their cis-acting elements. Our study concentrated on the promoter regions within 2000 bp upstream of the *PbbZIP* genes. It revealed that these genes possess diverse cis-acting elements, indicating their involvement in various plant functions. A total of 18 distinct cis-acting elements were identified, most of which are related to hormone regulation, light responses, abiotic stress resistance, and control of the circadian rhythm. For instance, these elements include ones that respond to hormones such as gibberellins, methyl jasmonate (MeJA), auxin, and abscisic acid. Notably, the promoters of *PbbZIP* genes exhibit a high frequency of light-responsive elements, which are present in most members of the gene family. Other common elements include those that respond to abscisic acid, hypoxia, and drought. The enrichment of these elements suggests that *PbbZIP* genes play significant roles in plant growth and development, hormone signal transduction, and responses to abiotic stresses.

**Figure 7:**
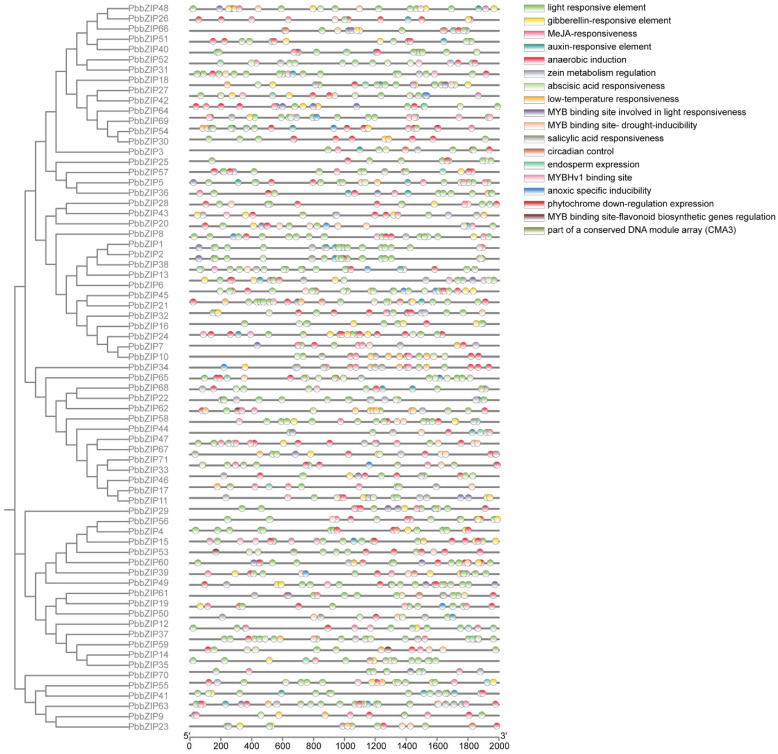
Predicted cis-acting elements in the promoter regions of *PbbZIP* genes. The one on the left is the promoter position (−2000 bp). The cis-acting regulatory elements in the promoter were categorized into 22 types, each represented by a different color. The lower axis denotes the quantity of each cis-acting element.

In this study, most of these genes contain MYB-binding sites. The MYB-binding site is implicated in light responsiveness and possesses several properties, such as drought inducibility, circadian control, salicylic acid responsiveness, light responsiveness, auxin responsiveness, and endosperm-specific expression. The MYBHv1-binding site, light reactivity, phytochrome down-regulation expression, anoxic-specific inducibility, light-reactive module, and light response elements are all cis-acting elements. The regulation of MYB-binding site flavonoid biosynthesis genes and members of a conserved DNA module array (CMA3) also contains one or more cis-acting element. These factors suggest that the expression of *PbbZIP* is linked to these abiotic stresses. (See Figure 7).

### 3.7. Expression Analysis of PbbZIP Genes in Different Tissues

**Figure 8:**
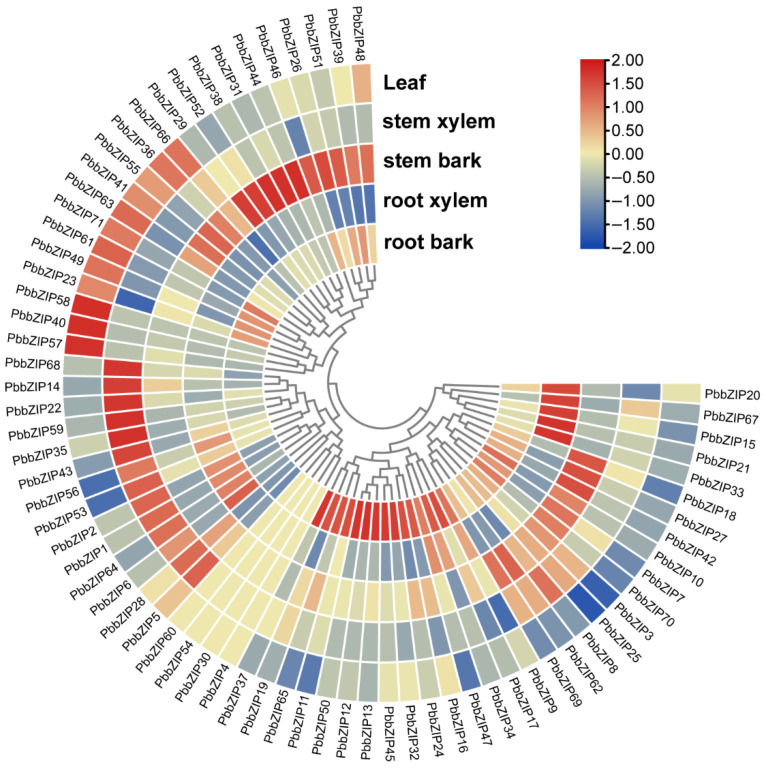
Tissue-specific gene expression patterns of 71 *PbbZIP* genes. The expression patterns of genes in the root bark, root xylem, stem bark, stem xylem, and leaf. The red and blue colors indicate the high and low transcript abundance, respectively.

To gain a more comprehensive understanding of the role and regulatory mechanism that *PbbZIPs* play in the growth and development of P. bournei, we investigated the expression patterns of the 71 *PbbZIP* genes (Figure 8). An analysis of gene expression heat maps revealed gene expression patterns specific to diverse tissues. *PbbZIPs* could be categorized into five distinct groups, which were highly expressed in root bark, root xylem, stem bark, stem xylem, and leaves (Figure 8). A greater number of *bZIP* genes were highly expressed in both root bark and stem bark, while relatively fewer were highly expressed in root xylem, stem xylem, and leaves. For instance, at least 24 *bZIPs* in the root bark group and 23 genes in the stem bark group predominantly exhibited elevated expression levels in root bark or stem bark. In contrast, there were considerably fewer genes, specifically only 14 or 15 *bZIPs*, which were highly expressed in the root xylem and stem xylem groups, respectively. Moreover, only 12 *genes* were highly expressed in leaves.

### 3.8. The Expression Profile of PbbZIP Genes Under Abiotic Stress

**Figure 9:**
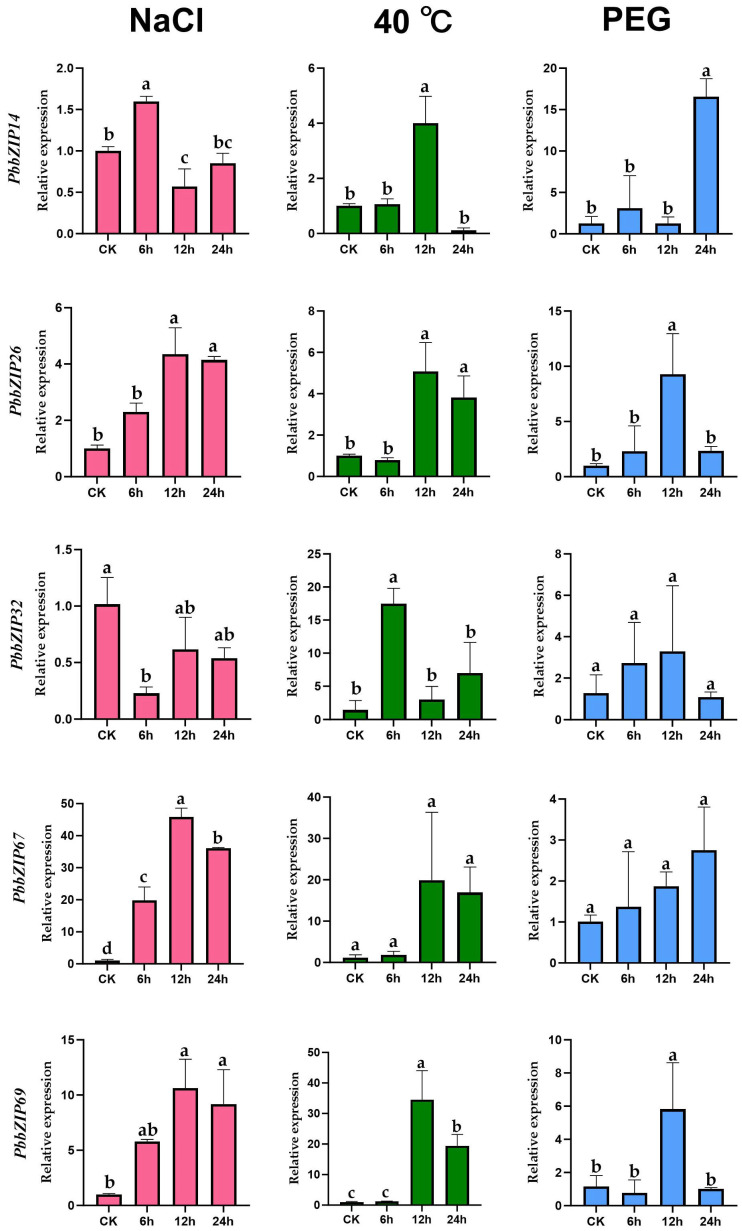
The relative expression levels of *PbbZIP* genes responding to abiotic stresses, as determined by RT-qPCR, were represented by pink for high salt, green for high temperature, and blue for drought stress, under each treatment at the same time points (0, 6, 12, and 24 h). Significances were indicated by different letters according to one-way ANOVA and Tukey’s multiple range tests (*p* < 0.05).

To explore how the *PbbZIP* gene family responds to non-biotic stresses such as salt, temperature, and drought, we selected five genes (*PbbZIP32*, *PbbZIP14*, *PbbZIP26*, *PbbZIP67*, and *PbbZIP69*) from the five *PbbZIP* subfamilies (A, D, E, I, and S) with the most members. These genes contain the highest number of adversity-related cis-acting elements in their respective subfamilies. Transcriptional analysis had verified that there were variable levels of transient expression in response to different stresses. Based on the results, *PbbZIP32*, *PbbZIP26*, *PbbZIP67*, and *PbbZIP69* exhibited significant up-regulation in response to salt stress induced by 10% NaCl. Specifically, the expression levels of *PbbZIP67* and *PbbZIP69* increased by more than 10-fold after salt treatment. In addition, the expression pattern of the RT-qPCR results indicated that the *PbbZIP* genes were subject to either up-regulation or down-regulation under the high temperature of 40 °C (Figure 9). More precisely, *PbbZIP32* reached its peak at 6 h, with its expression increasing by more than 15-fold. Analogous to its response to salt, the expression levels of *PbbZIP67* and *PbbZIP69* demonstrated a robust reaction to heat, with increases of nearly 20-fold or greater. Meanwhile, *PbbZIP14*, *PbbZIP67,* and *PbbZIP69* peaked at 12 h, while *PbbZIP26* peaked at 24 h. After attaining their respective peaks, all of these began to decline. Surprisingly, *PbbZIP14*, *PbbZIP67,* and *PbbZIP69* also showed similar patterns with significantly elevated expression under drought stress induced by 10% PEG. *PbbZIP14* demonstrated a robust response to PEG-induced drought stress, as its expression increased by more than 15-fold. 

## 4. Discussion

In this study, we systematically analyzed the *bZIP* gene family in *P. bournei*, revealing its crucial role in mitigating environmental stress. *P. bournei*, a subtropical evergreen tree of ecological and economic importance, is highly vulnerable to temperature changes and water scarcity in terms of growth and metabolism [51]. Under drought stress, it activates defense mechanisms, such as regulating osmotic substances and protective enzymes [36]. Previous research results indicate that the *bZIP* transcription factor family plays a crucial role in regulating both plant growth and development, as well as in response to abiotic stress [17,18,52]. Unlike the well-studied *bZIP* family in model plants such as *Arabidopsis* [9,53], the *P. bournei bZIP* family has been less explored. This study bridges this knowledge gap and offers fresh perspectives on how *P. bournei* adapts to stress. It also highlights the significance of gene regulation as a plant adaptation mechanism to environmental stress [54], laying a solid foundation for future research.

The number of *bZIP* genes varies among different plant species. For example, 78, 89, 125, 88, 86, 45, and 52 *bZIP* genes have been identified in *A. thaliana*, *O. sativa*, *Z. mays*, *J. regia*, poplar, *Z. jujuba*, and *C. tinctorius*, respectively. These genes may exhibit similar or diverse responses to various stresses. Based on the phylogenetic reconstruction (Figure 3A) using the Arabidopsis classification as a standard, *PbbZIPs* were divided into 12 subfamilies (A-K, S). Protein structure analysis also supported this classification. Notably, no PbbZIP proteins were found in the M subfamily, which may indicate that these proteins were lost during the evolution of *P. bournei*. The PbbZIP proteins exhibited a wide variety of physicochemical properties, which underscored their potential role in adapting to stresses, particularly under conditions such as salt stress, high temperature, and drought in *P. bournei*. These proteins varied in size, with the number of amino acid residues ranging from 74 to 839, leading to molecular weights ranging from 8813.28 Da to 88,864.94 Da. Their predicted subcellular localization, which was predominantly in the nucleus, was consistent with their function as transcription factors, indicating that they were involved in gene regulatory processes. The theoretical isoelectric points of these proteins ranged from 4.78 to 11.45, indicating diverse acidic and basic properties that could impact their stability and interactions with different cellular environments. The aliphatic index values, ranging from 47 to 94, reflected the various levels of thermostability of these proteins. The negative values of the Grand Average of Hydropathy (GRAVY) indicated their hydrophilic nature, which is closely related to their function in the aqueous cellular environments. Collectively, these diverse biophysical properties suggest that the *PbbZIP* genes likely play distinct regulatory roles in response to abiotic stresses. Similarly to the findings in other plant species, these results were consistent with those reported in a previous study [55], which found that 62 *bZIP* genes in Chinese pear have evolved through duplication, possess stress-responsive cis-elements, and exhibit differential hormonal expression under hormonal regulation.

Additionally, the alignment of PbbZIP protein sequences offers profound insights into their functional similarities and evolutionary relationships. By highlighting both the conserved regions and the variances within the protein sequences of the PbbZIP family, it enables a more in-depth understanding of these aspects (Figure 2). Interestingly, a conserved phosphorylation motif, “RHx[R/H]SxS”, has been shown to play a crucial role in *the Arabidopsis response to abiotic stress* [22,49,56]. Likewise, a conserved “NRxSA[R/K]RSRxRK” motif contains potential phosphorylation sites “S” as well. The research on *bZIPs* in *Arabidopsis* offers significant clues for uncovering the molecular mechanism through which *bZIPs* in *P. bournei* respond to external stimuli. The phylogenetic and homologous analyses of the *bZIP* transcription factor family in *P. bournei* and *A. thaliana* focus on their conservation and cross-species divergence, revealing the evolutionary relationships among bZIP proteins in these species. The collinearity suggested that the *bZIP* transcription factors that mediated stress responses in plant species were functionally similar and that the *bZIP* genes of these species were evolutionarily conserved [57]. Our results were identical to those of Tian [58], who demonstrated through phylogenetic analysis of NtNF-Y genes that revealed stress-responsive expression and diverse gene structures in tobacco.

During gene-family evolution, duplication events—whole-genome duplication (WGD), tandem duplication (TD), segmental duplication (SD), and retrotransposition (TRD)—serve as the primary engines of genomic innovation [59]. Among these, TD and SD are the dominant forces that shape family size and functional diversity [60,61]. Subsequent gene loss and selective amplification further sculpt divergent family architectures between herbaceous and woody lineages [62,63]. In *Arabidopsis*, for example, SD has been a major driver of *bZIP* expansion [8,9]. A comparable pattern is evident in *P. bournei,* where widespread SD has significantly increased the number of *PbbZIP* loci. Comparative synteny identified 45 high-confidence orthologous pairs between *P. bournei* and *Arabidopsis*, implying an unexpectedly close evolutionary relationship. This pronounced collinearity contrasts with the anticipated divergence between monocots and eudicots and likely reflects lineage-specific *loss of bZIP genes* in *P. bournei* after the shared paleopolyploidy events.

Members of the same subfamily exhibit a consistent pattern in motif composition and arrangement (Figure 4), consistent with their evolutionary characteristics. However, they show significant variation in exon–intron structures (Figure 6), implying divergent expression regulation mechanisms [44,50]. Notably, genes such as *PbbZIP17* and *PbbZIP54* lack a 5′-UTR. This is likely due to limitations in genome annotation. Tissue expression analysis revealed that 71 *PbbZIP* genes showed significant differences in expression intensity across the root periderm, root xylem, stem periderm, stem xylem, and leaves (Figure 8), indicating that they have tissue-specific functions and roles in regulating growth and development. This aligns with findings from research on the *LBD* gene family in *P. bournei* [53], where 38 family members were identified with tissue-specific functions related to root formation, light response, and stress adaptation.

Our findings align with the research of Guo [64] and Manzoor [55], who indicate that the bipartite network architecture helps stabilize intracellular protein concentration. This study analyzed the cis-acting elements in *PbbZIP* gene promoters and identified numerous regulatory motifs associated with hormone response, stress signaling, light reactions, and growth-related processes (e.g., auxin, abscisic acid, gibberellins, MeJA, low temperature, and anaerobic induction) (Figure 7). Notably, MYB-binding sites, which are key to light response, regulate circadian rhythms, drought resistance, salicylic acid response, and flavonoid biosynthesis via the CMA3 module, as well as phytochrome signaling. This finding is similar to those of Tang et al. [65] on 19 *bZIP* transcription factors in Magnaporthe oryzae, underscoring the conserved yet diverse roles of the *bZIP* family in stress responses and developmental regulation.

Previous studies have demonstrated that the *bZIP* gene family plays a crucial role in responding to various stressors, including salt and drought. For example, in Glycine max, overexpression of *bZIP2* enhances drought and salt tolerance by activating the transcription of *GmMYB48*, *GmWD40*, *GmDHN15*, and *GmLEA* [66]. In maize, *bZIP* transcription factors (TFs) interact with HsF08 to regulate responses to salt and drought stress, and activate genes such as *ZmDREB2A*, *ZmNCED*, *ZmERD1*, *ZmRD20*, and *ZmRAB18* [67]. Through qRT-PCR analysis, we have provided strong evidence of the regulatory role of *PbbZIP* genes in mitigating the adverse effects of salt stress. When salt stress was imposed using a 10% NaCl treatment, genes such as *PbbZIP32*, *PbbZIP26*, *PbbZIP67*, and *PbbZIP69* showed significant up-regulation. Notably, the expression levels of *PbbZIP67* and *PbbZIP69* increased more than ten-fold following treatment. This substantial induction of gene expression suggests that they may be involved in the activation of stress-response pathways.

In this study, we conducted a systematic analysis of the *bZIP* gene family in *P. bournei*, employing qRT-PCR to determine and validate the expression of *PbbZIP* genes under various abiotic stress conditions. Our findings not only shed light on the potential roles of *PbbZIP* in the physiological responses of *P. bournei* to heat, salt, and drought stresses but also provide fundamental insights into the molecular mechanisms underlying these responses. By revealing the involvement of *PbbZIP* genes in stress tolerance, this study lays the groundwork for future research aimed at enhancing the stress resilience of *P. bournei* through genetic engineering approaches.

## 5. Conclusions

In this study, we comprehensively investigated the *PbbZIP* genes in *P. bournei*, identifying 71 members through genomic data and computational methods. Primarily located in the nucleus of the cell, these genes play crucial roles in plant growth, development, and responses to stress. Our analysis of the physicochemical properties of PbbZIP proteins revealed a wide range of isoelectric points and conserved motifs, which indicated functional diversification within the family. This suggests that different members may perform distinct functions in plant biological processes. The chromosomal distribution of *PbbZIP* genes revealed frequent gene clusters in specific regions, with tandem and segmental duplications making a significant contribution to the family’s expansion. The expression patterns of these genes, particularly in stem and root tissues, indicate their essential role in plant development. RT-qPCR analyses demonstrated that *the expression levels of PbbZIP67 and PbbZIP69* increased significantly under saline conditions, highlighting their role in salt stress tolerance. Additionally, five genes—*PbbZIP14*, *PbbZIP26*, *PbbZIP32*, *PbbZIP67*, and *PbbZIP69*—showed enhanced expression in response to both salinity and heat stress. Although *PbbZIP67* and *PbbZIP69* seem to be specifically involved in salt and heat stress tolerance, other genes also respond to heat and salt stress, although the effect is relatively small under drought stress. These findings provide a solid foundation for further research. For instance, gene knockout experiments could further clarify the specific roles of *PbbZIP* genes in stress adaptation and resilience. This study provides valuable insights into P. bournei breeding and identifies key *bZIP* genes that can enhance resistance to temperature fluctuations, drought, and salinity. Overall, our results lay the foundation for future investigations into the impacts of abiotic stress on woody plants.

## Figures and Tables

**Table 1:** List of internal reference genes and primers used.

Gene Name	Forward Primer (5′-3′)	Reverse Primer (5′-3′)
*PbZIP14*	GCAGCCTGGTGAGGTAGC	GTGGACGTGGGGTAAGGC
*PbZIP26*	AGAGGTCCGAGTCCGCAA	CGTTCAACCCTTCCGCCT
*PbZIP32*	GACGAGCACCACAGGCAT	TCGGACTTGGCGGCAATT
*PbZIP67*	TCGGCATGCCTGATGGTG	TGACTCAGAGTCCGCGGA
*PbZIP69*	CATGGCCCCTGCAAGTGT	GGCACCACTCCACTTGCT
*PbEF1α*	CATTCAAGTATGCGTGGGT	ACGGTGACCAGGAGCA

**Table 2:** Detailed information on 71 *PbbZIP* Genes of *P. bournei* and their encoded proteins.

Gene Accession	ID	AA/aa	MW/Da	Theoretical pI	Aliphatic Index	GRAVY	Subcellular Localization
*OF19974-RA*	*PbbZIP1*	399	41,600.65	5.89	51.93	−0.67	Nucleus
*OF19973-RA*	*PbbZIP2*	144	16,506.95	6.28	82.64	−0.585	Nucleus
*OF19914-RA*	*PbbZIP3*	136	15,566.31	9.52	55.96	−1.396	Nucleus
*OF10270-RA*	*PbbZIP4*	231	25,909.15	8.5	58.74	−0.698	Nucleus
*OF18209-RA*	*PbbZIP5*	198	22,954.6	6.71	72.47	−0.855	Nucleus
*OF02523-RA*	*PbbZIP6*	141	16,414.99	7.8	82.34	−0.684	Nucleus
*OF11694-RA*	*PbbZIP7*	159	18,344.72	10.29	76.73	−0.76	Nucleus
*OF11818-RA*	*PbbZIP8*	325	36,545.42	9.25	69.05	−0.674	Nucleus
*OF11930-RA*	*PbbZIP9*	199	23,154.87	6.05	79.4	−0.855	Nucleus
*OF28301-RA*	*PbbZIP10*	144	16,675.1	6.19	75.83	−0.704	Nucleus
*OF28225-RA*	*PbbZIP11*	447	49,195.23	6.28	78.68	−0.46	Nucleus
*OF04812-RA*	*PbbZIP12*	327	36,606.14	8.68	64.68	−0.878	Nucleus
*OF04669-RA*	*PbbZIP13*	368	41,352.8	6.73	80.92	−0.451	Nucleus
*OF06905-RA*	*PbbZIP14*	322	35,902.66	7.96	79.97	−0.577	Nucleus
*OF08770-RA*	*PbbZIP15*	74	8813.28	10.34	59.46	−1.295	Nucleus
*OF03422-RA*	*PbbZIP16*	373	41,747.12	6.88	63.06	−0.914	Nucleus
*OF22080-RA*	*PbbZIP17*	169	19,451.17	9.23	70.89	−0.337	Nucleus
*OF12918-RA*	*PbbZIP18*	374	41,217.7	5.51	70.43	−0.613	Nucleus
*OF09866-RA*	*PbbZIP19*	387	42,269.25	6.38	61.4	−0.716	Nucleus
*OF05546-RA*	*PbbZIP20*	433	45,888.29	9.01	64.09	−0.659	Nucleus
*OF25842-RA*	*PbbZIP21*	368	40,364.2	8.74	57.61	−0.713	Nucleus
*OF24005-RA*	*PbbZIP22*	365	41,331.5	8.37	83.64	−0.368	Nucleus
*OF23680-RA*	*PbbZIP23*	277	31,093.66	5.51	76.03	−0.716	Nucleus
*OF23482-RA*	*PbbZIP24*	496	55,948.95	6.42	81.47	−0.393	Nucleus
*OF23448-RA*	*PbbZIP25*	461	52,301.17	5.78	76.68	−0.558	Nucleus
*OF10424-RA*	*PbbZIP26*	339	38,460.28	4.78	81.36	−0.556	Nucleus
*OF01790-RA*	*PbbZIP27*	117	13,424.64	6.72	90.85	−0.318	Nucleus
*OF01865-RA*	*PbbZIP28*	135	15,157	6.19	67.19	−0.977	Nucleus
*OF01905-RA*	*PbbZIP29*	324	35,867.17	7.19	65.12	−0.762	Nucleus
*OF01159-RA*	*PbbZIP30*	419	45,724.98	7.83	74.61	−0.562	Nucleus
*OF00429-RA*	*PbbZIP31*	244	26,831.46	5.51	67.99	−0.641	Nucleus
*OF29773-RA*	*PbbZIP32*	374	39,382.74	5.96	47.83	−0.74	Nucleus
*OF27741-RA*	*PbbZIP33*	470	51,343.31	6.89	76.47	−0.514	Nucleus
*OF15721-RA*	*PbbZIP34*	337	37,502.44	7.03	68.31	−0.788	Nucleus
*OF15812-RA*	*PbbZIP35*	217	24,620.4	7.03	76.45	−0.745	Nucleus
*OF11162-RA*	*PbbZIP36*	597	65,168.15	8.83	56.52	−0.898	Nucleus
*OF10986-RA*	*PbbZIP37*	280	29,704.17	5.45	67.93	−0.514	Nucleus
*OF10903-RA*	*PbbZIP38*	121	14,142.05	8.54	57.27	−1.001	Nucleus
*OF09410-RA*	*PbbZIP39*	139	16,192.43	6.83	73.74	−0.831	Nucleus
*OF09226-RA*	*PbbZIP40*	363	39,949.87	6.19	74.71	−0.599	Nucleus
*OF07945-RA*	*PbbZIP41*	389	42,749.74	5.97	68.71	−0.666	Nucleus
*OF05157-RA*	*PbbZIP42*	284	31,775.42	6.51	69.75	−0.744	Nucleus
*OF05208-RA*	*PbbZIP43*	468	52,189.65	6.52	74.49	−0.58	Nucleus
*OF05212-RA*	*PbbZIP44*	416	45,110	8.78	59.59	−0.779	Nucleus
*OF19397-RA*	*PbbZIP45*	407	46,959.1	8.7	63.51	−0.926	Nucleus
*OF19394-RA*	*PbbZIP46*	177	20,625.17	6.44	80.96	−0.801	Nucleus
*OF18693-RA*	*PbbZIP47*	167	18,448.37	9.74	60.78	−1.117	Nucleus
*OF18548-RA*	*PbbZIP48*	150	17,606.19	11.45	94.87	−0.687	Nucleus`
*OF28795-RA*	*PbbZIP49*	453	50,318.03	6.55	78.9	−0.482	Nucleus
*OF26318-RA*	*PbbZIP50*	151	17,332.69	6.59	79.47	−0.643	Nucleus
*OF26807-RA*	*PbbZIP51*	143	16,152.73	9.71	83.15	−0.45	Nucleus
*OF24581-RA*	*PbbZIP52*	235	25,001.1	9.56	57.74	−0.487	Nucleus
*OF06079-RA*	*PbbZIP53*	584	64,394.12	6.65	58.18	−0.9	Nucleus
*OF29662-RA*	*PbbZIP54*	273	29,723.97	6.55	62.89	−0.756	Nucleus
*OF16038-RA*	*PbbZIP55*	169	19,301.84	9.62	61.83	−0.872	Nucleus
*OF02992-RA*	*PbbZIP56*	376	42,234.62	7.68	60.51	−0.903	Nucleus
*OF03083-RA*	*PbbZIP57*	431	46,862.33	9.61	63.34	−0.757	Nucleus
*OF03232-RA*	*PbbZIP58*	262	29,182.16	9.59	47.33	−0.919	Nucleus
*OF01374-RA*	*PbbZIP59*	430	46,845.64	5.57	64.02	−0.78	Nucleus
*OF10634-RA*	*PbbZIP60*	170	20,349.2	7.74	68.82	−1.127	Nucleus
*OF29018-RA*	*PbbZIP61*	403	43,556.63	6.61	56.23	−0.771	Nucleus
*OF28905-RA*	*PbbZIP62*	358	39,138.26	6.5	62.23	−0.794	Nucleus
*OF18055-RA*	*PbbZIP63*	265	29,261.84	6.38	60.75	−0.774	Nucleus
*OF20655-RA*	*PbbZIP64*	348	38,339.97	5.89	62.61	−0.615	Nucleus
*OF20635-RA*	*PbbZIP65*	419	45,891.11	5.39	63.56	−0.692	Nucleus
*OF14232-RA*	*PbbZIP66*	260	28,706.32	6.82	71.69	−0.58	Nucleus
*OF17740-RA*	*PbbZIP67*	366	41,515.21	8.39	80.25	−0.485	Nucleus
*OF17491-RA*	*PbbZIP68*	180	19,918.45	8.68	71.56	−0.411	Nucleus
*OF12212-RA*	*PbbZIP69*	409	43,379.93	6.18	49.24	−0.838	Nucleus
*OF07610-RA*	*PbbZIP70*	448	48,779.58	7.18	78.04	−0.485	Nucleus
*OF26090-RA*	*PbbZIP71*	839	88,864.94	6.41	68.1	−0.464	Nucleus

Note: AA/aa: number of amino acids; MW/Da: molecular weight; pI: theoretical isoelectric point; Aliphatic Index: aliphatic index; GRAVY: grand average of hydropathicity.

**Table 3:** Synteny information and subfamily classification of *bZIP* genes in 45 groups of *P. bournei* and *A. thaliana*.

*A. Thaliana* Gene ID	*A. Thaliana* Gene Name	CLASS	*P. bournei* Gene ID	*P. bournei* Gene Name	CLASS
*AT5G49450*	*ATbZIP1*	S	*OF08770-RA*	*PbbZIP15*	S
*AT5G49450*	*ATbZIP1*	S	*OF06079-RA*	*PbbZIP53*	S
*AT2G18160*	*ATbZIP2*	S	*OF09410-RA*	*PbbZIP39*	S
*AT5G15830*	*ATbZIP3*	S	*OF15812-RA*	*PbbZIP35*	S
*AT3G49760*	*ATbZIP5*	S	*OF04812-RA*	*PbbZIP12*	S
*AT3G49760*	*ATbZIP5*	S	*OF10986-RA*	*PbbZIP37*	S
*AT2G22850*	*ATbZIP6*	S	*OF04812-RA*	*PbbZIP12*	S
*AT2G22850*	*ATbZIP6*	S	*OF10986-RA*	*PbbZIP37*	S
*AT4G37730*	*ATbZIP7*	S	*OF04812-RA*	*PbbZIP12*	S
*AT4G37730*	*ATbZIP7*	S	*OF10986-RA*	*PbbZIP37*	S
*AT4G34590*	*ATbZIP11*	S	*OF09410-RA*	*PbbZIP39*	S
*AT4G35900*	*ATbZIP14*	A	*OF24005-RA*	*PbbZIP22*	A
*AT4G35900*	*ATbZIP14*	A	*OF05212-RA*	*PbbZIP44*	A
*AT4G35900*	*ATbZIP14*	A	*OF17491-RA*	*PbbZIP68*	A
*AT5G42910*	*ATbZIP15*	A	*OF17740-RA*	*PbbZIP67*	A
*AT2G40950*	*ATbZIP17*	B	*OF23448-RA*	*PbbZIP25*	B
*AT2G40620*	*ATbZIP18*	I	*OF12918-RA*	*PbbZIP18*	I
*AT4G35040*	*ATbZIP19*	F	*OF01865-RA*	*PbbZIP28*	F
*AT4G35040*	*ATbZIP19*	F	*OF05208-RA*	*PbbZIP43*	F
*AT5G06950*	*ATbZIP20*	D	*OF10903-RA*	*PbbZIP38*	D
*AT1G22070*	*ATbZIP22*	D	*OF25842-RA*	*PbbZIP21*	D
*AT2G16770*	*ATbZIP23*	F	*OF01865-RA*	*PbbZIP28*	F
*AT2G16770*	*ATbZIP23*	F	*OF05208-RA*	*PbbZIP43*	F
*AT2G17770*	*ATbZIP27*	A	*OF24005-RA*	*PbbZIP22*	A
*AT2G17770*	*ATbZIP27*	A	*OF17491-RA*	*PbbZIP68*	A
*AT4G38900*	*ATbZIP29*	I	*OF01790-RA*	*PbbZIP27*	I
*AT4G38900*	*ATbZIP29*	I	*OF05157-RA*	*PbbZIP42*	I
*AT2G21230*	*ATbZIP30*	I	*OF01790-RA*	*PbbZIP27*	I
*AT2G21230*	*ATbZIP30*	I	*OF05157-RA*	*PbbZIP42*	I
*AT2G42380*	*ATbZIP34*	E	*OF00429-RA*	*PbbZIP31*	E
*AT3G19290*	*ATbZIP38*	A	*OF17740-RA*	*PbbZIP67*	A
*AT4G36730*	*ATbZIP41*	G	*OF16038-RA*	*PbbZIP55*	H
*AT1G75390*	*ATbZIP44*	S	*OF09410-RA*	*PbbZIP39*	S
*AT5G65210*	*ATbZIP47*	D	*OF19397-RA*	*PbbZIP45*	D
*AT3G56660*	*ATbZIP49*	B	*OF23448-RA*	*PbbZIP25*	B
*AT1G77920*	*ATbZIP50*	D	*OF19397-RA*	*PbbZIP45*	D
*AT3G62420*	*ATbZIP53*	S	*OF08770-RA*	*PbbZIP15*	S
*AT3G62420*	*ATbZIP53*	S	*OF06079-RA*	*PbbZIP53*	S
*AT3G62420*	*ATbZIP53*	S	*OF10634-RA*	*PbbZIP60*	S
*AT5G11260*	*ATbZIP56*	H	*OF11162-RA*	*PbbZIP36*	H
*AT5G10030*	*ATbZIP57*	D	*OF25842-RA*	*PbbZIP21*	D
*AT3G17609*	*ATbZIP64*	H	*OF03083-RA*	*PbbZIP57*	H
*AT5G06839*	*ATbZIP65*	D	*OF02523-RA*	*PbbZIP6*	D
*AT5G06839*	*ATbZIP65*	D	*OF19974-RA*	*PbbZIP1*	D
*AT1G32150*	*ATbZIP68*	G	*OF18055-RA*	*PbbZIP63*	G

## Data Availability

Data are contained within the article.
